# The Association Between Serum Homocysteine Levels and Placenta-Mediated Complications: A Narrative Review

**DOI:** 10.7759/cureus.31305

**Published:** 2022-11-09

**Authors:** Sharmeen I Memon, Neema S Acharya

**Affiliations:** 1 Department of Obstetrics and Gynaecology, Jawaharlal Nehru Medical College, Datta Meghe Institute of Medical Sciences, Wardha, IND

**Keywords:** pre-eclampsia, placenta-mediated complications, fetal growth restriction, recurrent pregnancy loss, hyperhomocysteinemia, homocysteine

## Abstract

The most extremely unfavourable outcome of pregnancy is the death of the mother and newborn. Negative outcomes for mothers or babies can occur as a result of complications or issues during pregnancy, birth or the post-partum period. Early elevated maternal homocysteine (Hct) levels during pregnancy have been linked to altered placental development. There is evidence that suggests an elevated maternal blood Hct level is the new obstetrical risk factor, and the association between hyperhomocysteinemia (HHct) and numerous obstetrical problems was recently recognised.

Hct is an essential amino acid, which contains sulphur and is formed from the metabolism of methionine. HHct has several known aetiologies, including genetic anomalies; a deficiency in folic acid, vitamin B6 and vitamin B12; hypothyroidism; old age; and renal illnesses. Vascular problems, coronary artery disease, atherosclerosis and embolic illnesses can all occur as a result of high blood levels of Hct. Hct levels are lower in normal pregnancies than it is in women who are not pregnant. Many pregnancy-related problems, including pre-eclampsia (PE), recurrent pregnancy loss (RPL), placental abruption, premature delivery and foetal growth restriction (FGR) have been connected to HHct in recent research.

We looked for pertinent literature using a thorough and systematic search from PubMed, Medline, Embase, Cochrane Library, Google, etc., and articles that were published before August 2022 based on serum Hct levels and various placenta-mediated complications for this review.

In this review, we described the synthesis and metabolism of Hct in humans, Hct levels at various phases of normal pregnancy and the association between Hct and placenta-mediated pregnancy complications.

The outcomes discovered can help obstetricians increase the likelihood of a successful pregnancy in cases where placenta-mediated issues are present. Lowering Hct levels with a high dose of folic acid tablets during the subsequent pregnancy may be useful for women who experienced these difficulties in prior pregnancies as a result of HHct.

## Introduction and background

Homocysteine (Hct) is a naturally occurring, sulphur-containing amino acid formed during methionine metabolism [1]. Metabolism of Hct involves two pathways:

1) Remethylation: Vitamin B12 and folate are required for the conversion of Hct to methionine by remethylation [2].

2) Transsulphuration: It is the process of removing sulphur from a substance. By the enzymatic action of cystathionine beta-synthase (CBS), which requires vitamin B6 as a cofactor, a portion of Hct is transsulphurated to cystathionine [3].

**Figure 1:**
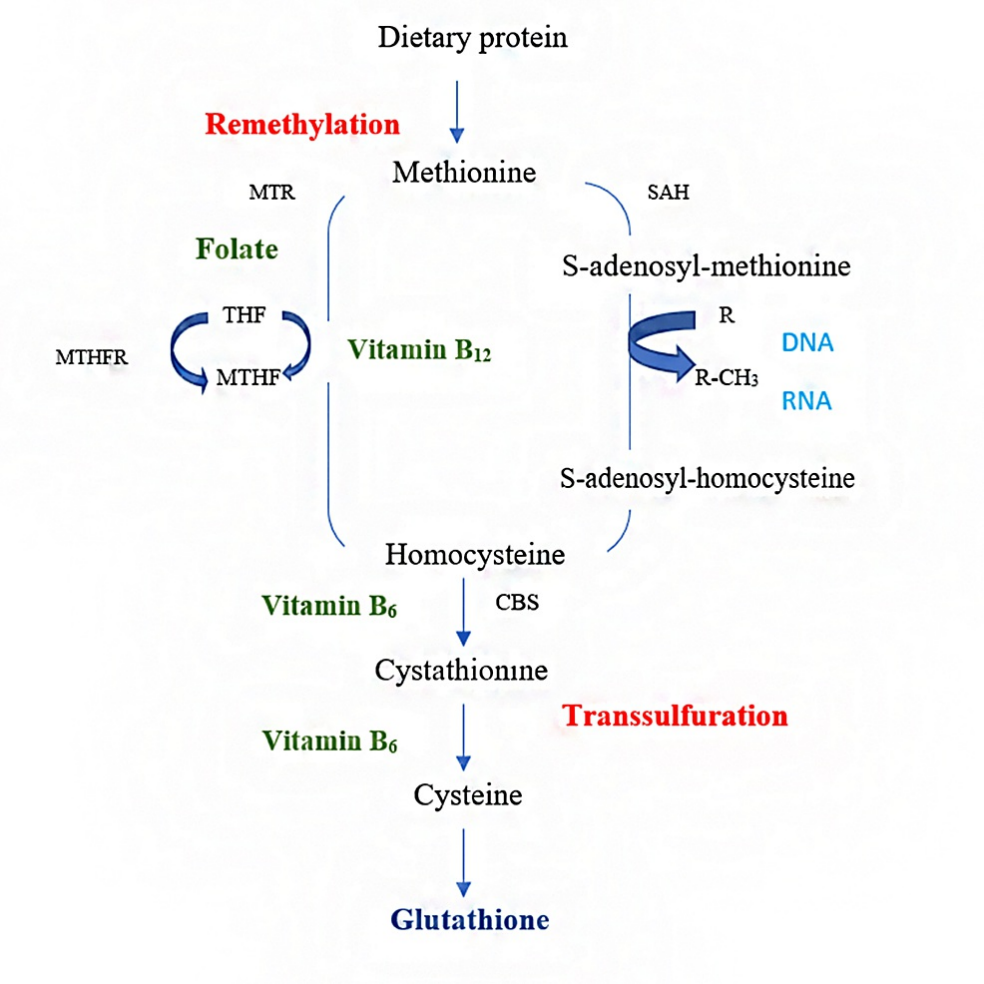
Methionine homocysteine cycle. CBS, cystathionine beta-synthase; MTR, methionine synthase; MTHF, methyltetrahydrofolate; MTHFR, 5,10-methylene tetrahydrofolate reductase; SAH, *S*-adenosylhomocysteine Figure credits: SIM and NSA

The majority of Hct, however, is remethylated to yield methionine. This reaction uses 5-methyltetrahydrofolate as a donor molecule in most tissues and vitamin B12 as a cofactor for the enzyme methionine synthase [4]. Adenosine triphosphate (ATP) transforms a fraction of methionine to *S*-adenosylmethionine (SAM). SAM acts as a methyl donor for a wide range of acceptors, such as DNA, RNA, lipids, proteins and hormones [5]. This is an important event in several metabolic pathways, including the methylation of DNA, which is vital for gene expression regulation. This methylation reaction results in SAM, which is rapidly eliminated by hydrolysis and produces Hct and adenosine. The regenerated Hct is then available to begin a new cycle of the methyl transfer group [6]. This reversible hydrolysis reaction favours the formation of *S*-adenosylhomocysteine (SAH; Figure 1).

Hct is not found in the human diet. Methionine is the only source of Hct. Methionine is a protein-forming amino acid that can be acquired in one of two ways: from diet or by remethylation of Hct [7]. It is mostly obtained from dietary protein, which includes animal proteins, and is also derived from proteins within the body, primarily as a result of muscle mass turnover. Hct's fate is determined by the availability of methionine in the diet. When methionine is insufficient, Hct is remethylated by transferring a methyl group from a donor molecule [8].

Folate, a member of the vitamin B complex, acts as a coenzyme by either absorbing or donating a carbon unit in several metabolic pathways. Folic acid's chemical name is pteroylmonoglutamic acid. Folate must be obtained through food sources as mammals cannot synthesise it [9]. Starting with serine as a carbon source and vitamin B6-dependent serine hydroxymethyltransferase enzymes, the folate cycle begins with the conversion of tetrahydrofolate to 5,10-methylene-THF. The primary role of folate is to transfer a single carbon atom, thereby facilitating cell division and DNA synthesis. Folate deficiency causes megaloblastic anaemia, reticulocytopenia, thrombocytopenia and leukopenia, all of which can be reversed by eating more folate-rich foods or taking a supplement. Regulating Hct degradation is primarily the function of the folate cycle [10]. Methyltetrahydrofolate is the only circulating folic acid form involved in the premethylation of Hct to methionine [11].

The metabolism of Hct in pregnancy is complicated, involving enzymes and a vitamin B complex. When any of these substances are deficient in supply or function, it can result in deleterious effects on homeostasis. In a non-pregnant female, normal Hct levels should be between 5 and 12 micromol/L. During pregnancy, hemodilution lowers Hct levels due to an increase in blood volume and a greater glomerular filtration rate [12]. Hct decreases early in pregnancy, reaches a minimum in the second trimester, and then gradually increases in the third and fourth trimesters to reach the levels seen in the first trimester. Before 16 weeks of pregnancy, Hct levels should be between 3.9 and 7.3 mmol/L; between 20 and 24 weeks of pregnancy, they should be between 3.5 and 5.3 mmol/L; and after 36 weeks of pregnancy, they should be between 3.3 and 7.5 mmol/L. In pregnancy, HHct refers to anything over this threshold (hyperhomocysteinemia) [13].

The presence of Hct levels that are more than the 90th or 95th percentile of the relevant reference group indicates HHct. HHct means your body is releasing an abnormal amount of Hct through its export system, which is a sign of a disturbance in Hct metabolism. Hydrogen peroxide and superoxide free radicals are produced during HHct. Poor feto-maternal outcomes will arise from the oxidative damage to endothelial cells, the decreased number of blood vessels in the villi and the diminished blood circulation at the feto-maternal interface that these stresses generate. Additionally, HHct promotes cell death and damage, leading to trophoblast dysfunction. Inhibition of nitric oxide release by endothelial cells, platelet aggregation and thrombosis promotion all result in activation of coagulation and endothelial damage, which reduces placental perfusion due to HHct [14].

Due to a lack of knowledge about the significance of serum Hct detection during pregnancy, many practitioners underestimate its importance. Recent studies have connected HHct to a range of pregnancy issues, including pre-eclampsia (PE), recurrent pregnancy loss (RPL), foetal growth restriction (FGR), preterm delivery, placental abruption and gestational diabetes mellitus (GDM) [15]. The purpose of this paper is to examine the current state of knowledge concerning Hct and its association with placenta-mediated pregnancy complications.

Search strategy

This study adhered to the principles of the *Preferred Reporting Items for Systematic Reviews and Meta-Analyses (PRISMA)*. The literature was searched using the online resources PubMed, Medline, Embase, the Cochrane Library and Google. Medical subject headings (MeSH) terms such as "homocysteine," "recurrent pregnancy loss," "preeclampsia," "placental abruption," "foetal growth restriction" and "pregnancy" were used to find relevant articles in PubMed. Each article was written in English and published between January 2000 and August 2022. All the papers selected for this study passed the following criteria: English language research papers are included; there are studies from the past 20 years; and the relationship between Hct and placenta-mediated problems during pregnancy has been the subject of discussion.

Selection criteria

Systematic reviews, randomised and non-randomised human-controlled trials, and retrospective and prospective cohort studies were included in the selection criteria. Studies included the correlation between serum Hct levels and placenta-mediated pregnancy complications. Animal studies, cadaver studies, in vitro studies, technical reports, editorials and review papers were excluded.

Study selection

**Figure 2:**
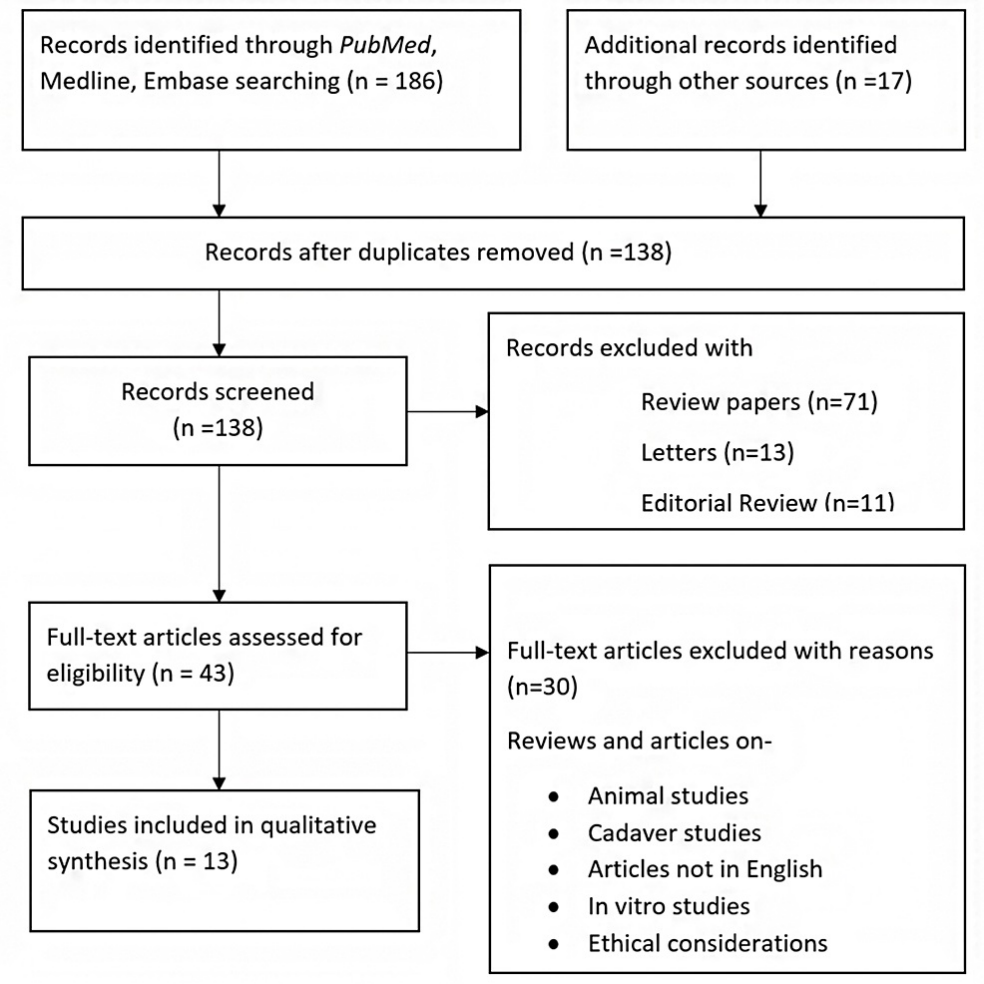
PRISMA flowchart of included studies. PRISMA, Preferred Reporting Items for Systematic Reviews and Meta-Analyses Figure credits: SIM and NSA

During the preliminary search, we found a total of 203 items that fit the criteria. Sixty-five studies were disqualified because they were duplicates, while another 95 were disqualified because they were not relevant. Thirty studies were disqualified after reviewing the entire text of the papers because they lacked key information. Finally, 13 studies met the requirements for inclusion in the meta-analysis (Figure 2).

## Review

Serum Hct levels in PE

Proteinuria and hypertension (blood pressure [BP] > 140/90 mmHg) are used to define PE after the 20th week of pregnancy [16]. Organs such as the heart, lungs, kidneys, liver, and brain are all negatively impacted by this disease. The exact cause is unknown; however, immunological abnormalities, endothelial dysfunction and inflammation are all plausible reasons [17].

Serum Hct levels were measured in a study of pregnant females, with both normotensive (n = 1,825) and hypertension (n = 401) cases included. PE was related to a 4.5% higher blood Hct level and a 1.6-fold higher risk of presenting elevated Hct than women with uncomplicated pregnancies [18]. Hct levels were elevated in women with a history of PE, years after giving birth. Moreover, the expression of high Hct levels is an excellent predictor of future cardiovascular disease [19].

Serum Hct levels in RPL

Two or more spontaneous abortions are considered RPL by the American Society for Reproductive Medicine, but three or more abortions are considered RPL by the European Society for Human Reproduction and Embryology (ESHRE) [20]. The morbidity rate of RPL is 5% in sexually active couples. Genetic conditions, chromosomal abnormalities, anatomical abnormalities, endocrine concerns and immunological disorders are just some of the potential causes of RPL [21].

Hereditary thrombophilic deficiencies, high Hct expressions or both are pathologic concerns in RPL that contribute to a propensity to venous thrombosis. About one-third of those with RPL also have HHct. As elevated Hct can injure endothelial cells, it may affect placental blood vessels and lead to RPL [22]. Insulin resistance (IR) and endocrine dysfunctions have been linked to an increase in inflammatory cytokines like tumour necrosis factor (TNF) and HHct. IR may cause abortion as it lowers blood flow to endothelial cells and degrades blood vessel integrity. Two important risk factors for RPL have been identified: low folate levels and high Hct levels [23].

Serum Hct levels in preterm delivery

The term *preterm delivery* describes the ending of a pregnancy earlier than 37 weeks of gestation due to a medical condition or unexpected labour. There are over 15 million preterm births worldwide each year [24]. Preterm birth can occur for a variety of reasons, such as genital abnormalities, systemic inflammation and other pregnancy difficulties. Recent research has established a link between preterm delivery and gene alterations associated with Hct metabolism. Hct may be linked to preterm labour because of its effects on endothelial cells, which may cause vascular blockage [25].

A comprehensive analysis of literature released between January 2000 and May 2020 was carried out. Three of the included papers covered the relationship between Hct and spontaneous preterm delivery [26]. According to two papers, a high amount of Hct throughout the second trimester and during delivery were both associated with early delivery. One study, however, found no connection between Hct expression in late pregnancy and preterm birth [27].

Serum Hct levels in placental abruption

Before delivery, there may be partial or complete placental segregation due to the breach of the spiral arteries of the uterus, which is referred to as placental abruption [28]. Clinical symptoms or signs are used to make the diagnosis of placental abruption. Abdominal pain and vaginal bleeding may be visibly present in patients with placental abruption. Pathological analysis of placental tissue after delivery confirms the diagnosis [29].

In a study, 100 healthy pregnant women (control group) who had at least one normal birth were compared with 46 women who were affected by placental abruption (experimental group). During the third trimester, blood was taken from all the pregnant women [30]. As a result, the experimental group's mean plasma Hct level was considerably greater than the control group. According to the study, high Hct may be a predictor of placental abruption [31].

Additionally, 7,587 participants were used in another study [32]. Hct was used as the first persistent variable in the authors' multivariable logistic regression analyses. The authors found that higher Hct was not linked to placental abruption after controlling the gestational weeks at the time of blood sampling and the general features of the participants [33]. The various definitions of HHct may be the cause of the variation in experimental outcomes [34].

Serum Hct levels in FGR

FGR is a condition in which a foetus fails to develop to its normal potential. A clinical definition of FGR is a birth weight that is less than the 10th percentile or 2 standard deviations below the average weight for the same gestational age or that does not surpass 2,500 g after 37 weeks of gestation [35]. FGR can be traced back to issues on the mother's end, issues with the placenta or umbilical cord, or problems with the own development of the foetus [36].

More than the 95th percentile of Hct was associated with an elevated incidence of FGR in a meta-analysis of 21,326 pregnant women across 19 studies [37]. When the Hct level is above the 90th percentile, the likelihood of having a baby that is small for their gestational age increases by 25%, according to this study [38]. However, some authors have argued that mothers who maintain a higher Hct during pregnancy are less likely to have babies affected by FGR. Then, there was some debate over the final result [39].

First, the authors claimed that the Hct tests were performed no later than 48 hours post-delivery [40]. The Hct levels may have altered dramatically 48 hours after delivery; hence, the figures may not reflect the true Hct level at delivery [41]. Ideally, blood samples for Hct testing should be taken before conception, during the first trimester, in the second trimester, at term, immediately following birth and one week later [42]. The statistical significance of using these numbers is much higher in confidence. Second, in the absence of precise statistical evidence, whether there was a significant difference in Hct levels between control neonates and experimental infants or between their mothers was questioned. Consequently, the unexpected findings on the connection between Hct and FGR require further evidence [43].

Serum Hct levels in GDM

GDM is a form of impaired glucose tolerance that develops or is initially identified while a woman is pregnant. IR and cell abnormalities are the two main metabolic issues that GDM patients are now experiencing [44]. These serve a crucial purpose in the genesis of GDM. According to certain studies, high Hct expression is positively correlated with the degree of IR. Therefore, the significance of plasma Hct in GDM has been highlighted in certain empirical research [45].

Five papers bolstered the association between Hct expressions and GDM, while four papers disproved the link [46]. Elevated Hct has been connected to GDM in some cross-sectional trials in Western countries, but not all of them. Serum Hct was substantially linked with glucose levels measured after two hours in GDM patients [47]. Serum Hct levels in GDM patients were higher than in healthy pregnant women between weeks 24 and 28 of pregnancy [48]. Sixty people with GDM and 19 healthy pregnant women were studied for a Polish study that examined their serum Hct levels. According to the results of the study, there was no distinct variation in Hct levels between the two groups. Glycosylated haemoglobin and fasting blood sugar levels were not linked with Hct [49].

Various disorders have been linked to elevated Hct levels. Multiple factors, both genetic and environmental, affect plasma Hct levels. Reducing Hct levels with treatment is both cost-effective and risk-free [50]. Therefore, it is crucial to maintain research efforts in this area to determine the processes through which HHct causes disease and the possible benefits of treatment [15].

## Conclusions

A wide range of research studies has concluded that HHct is responsible for endothelial damage and dysfunction and has been linked to multiple adverse pregnancy outcomes, including, but not limited to, PE, premature delivery, abruptio placentae, RPL, FGR and GDM. Although estimating serum Hct levels is an easy test, further large-scale studies are required before it can be recommended as a predictive marker for placenta-mediated complications during pregnancy. Understanding the correlation between Hct and human pregnancies entails prospective trials, ideally beginning before conception and continuing through all stages of pregnancy and into the post-partum period. Prenatal and antenatal vitamin B12 and folate supplementation decreases maternal and foetal morbidity and mortality through improving endothelial function and decreasing Hct levels. Women who have already experienced complications related to HHct may benefit from taking large doses of folic acid and vitamin B12 supplements to prevent further issues during future pregnancies.
